# Numerical Analysis of Stapes Prosthesis Constraining in the Case of Otosclerosis

**DOI:** 10.3390/ma14247747

**Published:** 2021-12-15

**Authors:** Virginija Gylienė, Valdas Eidukynas, Giedrius Gylys, Shalini Murugesan

**Affiliations:** 1 Faculty of Mechanical Engineering and Design, Kaunas University of Technology, Studentų Str. 56, 51424 Kaunas, Lithuania; valdas.eidukynas@ktu.lt (V.E.); shalini.murugesan@ktu.edu (S.M.); 2 Department of Otolaryngology, Lithuanian University of Health Sciences, Eivenių Str. 2, 44307 Kaunas, Lithuania; giedrius.gylys@kaunoklinikos.lt

**Keywords:** stapes prosthesis, adjustable angled prosthesis, incus long process, footplate, otosclerosis, eigenfrequency analysis

## Abstract

In the case of otosclerosis, it has been noticed that even though there are a variety of different prostheses on the market, due to the anatomical characteristics, it is not always possible to restore excellent mobility to the ossicles and the prosthesis. On the one hand, this happens because the incus long process and the prosthesis create difficult angles. On the other hand, incus necrosis is among the most common causes of the loss of stability to the prosthesis and stapedectomy failure. The aim of this research is to suggest an improvement for stapes prosthesis stability and numerically evaluate the impact of the prosthesis constraining to its dynamical behavior. Numerical 3D models of a standard as well as a modified (adjustable angled) stapes prosthesis were created in order to achieve this aim. Consequently, the modal analysis has been performed to evaluate the mechanical behavior of the prosthesis, assuming that the piston (thick part) would be made of Teflon, and the thin part, fixated on the incus long process, would be made from titanium alloy. Finally, the numerical analysis has been conducted by changing the boundary conditions in respect of the prosthesis constraining, where the attached stapes prosthesis connects to the ossicular chain. Subsequently, there were two hypotheses raised for the prosthesis loop constraining. The first is that during the surgery, the prosthesis is perfectly crimped with certain looseness. The second is that the prosthesis is outgrown by the tissues over time and thus becomes over-constrained. Then, the analyzed standard stapes prosthesis does not fulfil its functions because of the over-constraining that develops over time. An improvement for the standard stapes prosthesis, i.e., a modified stapes prosthesis (adjustable angled), that has been proposed in this research allows avoidance of the negative effects of the over-constrained standard stapes prosthesis that appear over time. Moreover, the proposed modified prosthesis helps to regain hearing when the angle between the incus long process and prosthesis is unfavorable.

## 1. Introduction

**Figure 1:**
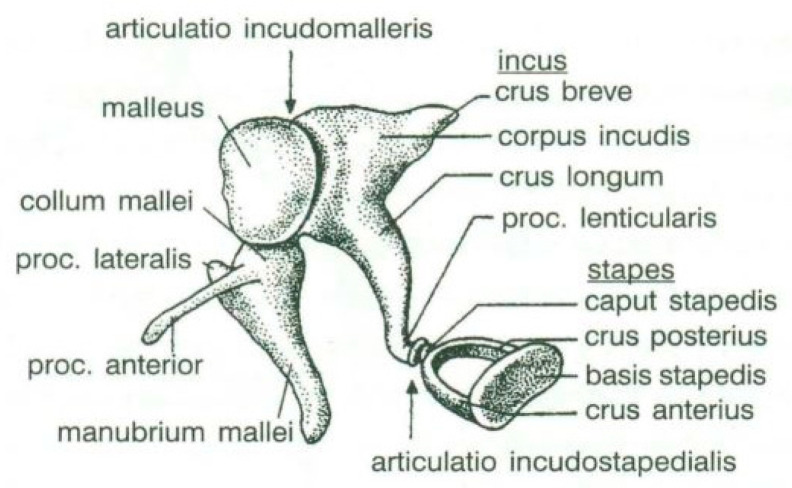
The ossicular chain of human ear. Source: adapted from Stropus et al. [2].

The human hearing capability is assured due to the conduction of mechanical vibrations along the ossicular chain to the inner ear [1]. The ossicular chain consists of three bones: the malleus, the incus, and the stapes (see Figure 1 [2]) that connects the tympanic membrane to the inner ear and plays an important role in amplifying and regulating sound waves [3].

The ossicular chain movements represent a key role in the process of hearing. The incus, the middle bone in the ossicular chain, is often reported in existing studies to be the most eroded bone and more distant from the cholesteatoma entrance [4,5]. Usually, the stapes, the smallest bone in the human body, is affected by otosclerosis, which is one of the most common causes of acquired hearing loss in the population [6]. Otosclerosis is a bone remodeling disorder in which the footplate of the stapes is replaced by an abnormal bone leading to an abnormal sound wave transmission to the inner ear at the level of the ear’s oval window [7]. Finally, in otosclerosis, the stapes becomes immobilized, and incus erosion leads to a progressive conductive [8] and mixed type hearing loss [9]. The only way to improve conductive hearing loss due to otosclerosis is with stapes surgery [10].

It has been noticed that stapes surgery can significantly improve the hearing of patients with otosclerosis [11]. Moreover, Huettenbrink and Beutner [6] revealed that the replacement of otosclerotic fixed stapes with a prosthesis is a highly successful surgical procedure. In order to achieve excellent and stable hearing results, stable and long-term attachment of the prosthesis to the incus long process is crucial [6]. The same statement was proved by Huber et al. [12], revealing that the hearing reconstruction necessitates ideal crimping of the prosthesis in order to obtain consistently good results.

Sim et al. [13] also revealed that, in the surgical treatment of otosclerosis, the coupling between the long process of the incus and the prosthesis is critical. Eiber et al. [14] emphasized that Van der Walls’s force, adhesion, and friction are involved in this coupling, which depends on the quality of the fixation (crimping) [13].

**Figure 2:**
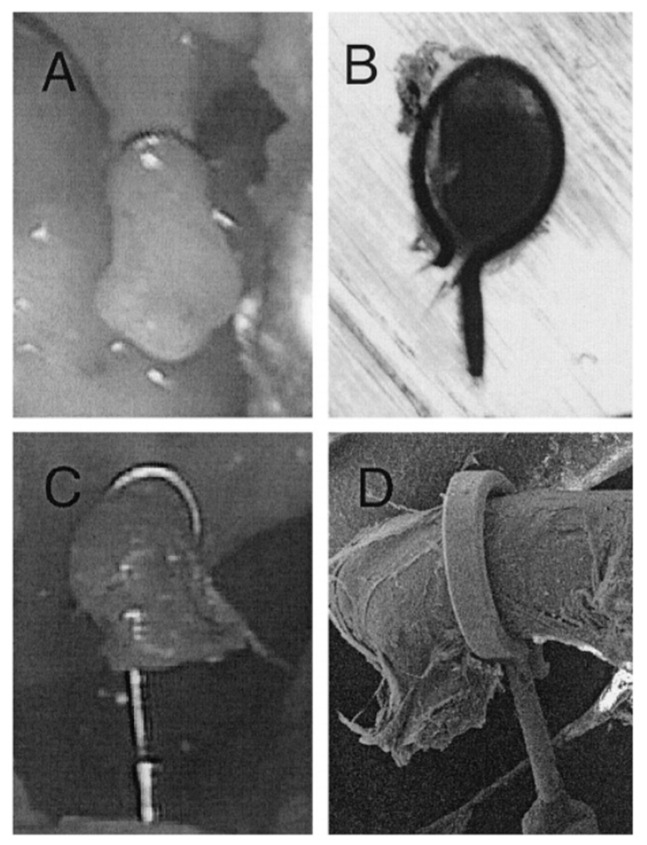
Morphological evaluation of the prosthesis loop. (**A**) Endoscopic and (**B**) micro grinding photography of tight crimping of fixing loop of prosthesis, (**C**) endoscopic and (**D**) scanning electron microscopy of tight crimping of fixing loop of prosthesis. Source: adapted from Huber et al. [12].

A typical stapes prosthesis consists of a cylindrical piston and an attachment, usually a wire or ribbon, designed to be crimped around the incus [15]. The visualization of the piston fixation was presented by Huber et al. [12] due to the endoscopy and microscopy, taken from different angles, and the tight and loose crimping. Figure 2 [12] shows a tight fixing loop of the prosthesis, which connects the incus and the footplate. As presented in Figure 2, situation (**A**,**B**) shows a too-tight connection of the long process of the incus, and (**C**,**D**) shows a normal connection between the prosthesis and the long process of the incus.

However, the final functional hearing gain depends on many different intraoperative and postoperative factors. For example, it has been revealed [16] that tight fixation of the stapes prosthesis yields better functional results because the sound transmission from the incus to the prosthesis is improved.

Despite that, the optimal prosthesis that should be used for otosclerosis surgery is still a matter of debate. The reason why nitinol prostheses may produce a better result is that the heat activation forms a tighter fixation rather than the manual crimping of the prosthesis loop [16].

It has been clinically proven that a too-tightly crimped or constrained prosthesis loop does not provide positive results as well as a not sufficiently crimped loop, i.e., a loose prosthesis. After numerous stapedectomy operations, it has been noticed that due to unknown reasons, when the prosthesis is fixated, a sulcus or hyperostosis is formed in the incus long process. Such a clinical situation has been observed when in the long-term, the placed stapes prosthesis leaves the ideal placement where it was constrained during the operation. Then, the prosthesis leans on the footplate with its edges; or in mechanical terms, it stretches tightly as a cord. In such a case, a revisional operation is necessary, during which the prosthesis is loosened and crimped again.

**Figure 3:**
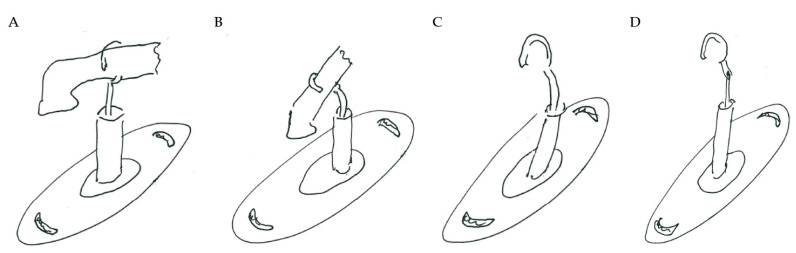
The visualization of stapes prosthesis problematics: ideal case study, problematics, and solution. (**A**) ideal/target placement of stapes prosthesis, (**B**) real placement of incus long process in respect of prosthesis (or obtained long-term after the operation), (**C**) view of prosthesis that has lost stability and is leaning not with the surface of the piston, (**D**) solution to achieve and retain the stapes prosthesis stability: prosthesis with the joint (adjustable angled prosthesis).

As it can be seen from the analyzed cases, the ability of stapes prosthesis to retain stability would allow avoidance of repeating (revisional) operations. It is thought that the stability of stapes is influenced by the fact that usually, in real cases, the angle between the incus long process and prosthesis does not form 90°, which would be an ideal or standard case. In real cases, when the stapes prosthesis is constrained, the placement between the incus long process and prosthesis changes, which results in a bent prosthesis, and its piston placement changes as well. Consequently, the ossicular chain stops transmitting sound. Figure 3 presents the recently described problem and its possible solution.

As it can be seen from Figure 3, an ideal or target procedure is fitted in situation (**A**), and sometimes the long process of the incus is on the uncomfortable angle of the procedure vector. In situations (**B**,**C**), facial nerve overhang is interfering with the operation vector. All the problems may be easily solved by using a modified stapes prosthesis, which is presented in Figure 3D.

In the previous chapter, the problematics of the stapes prosthesis stability after the stapes surgery due to otosclerosis was revealed. On the one hand, the stapes prosthesis stability depends on its successful placement during the surgery operation, which in fact depends on the anatomy of hearing ossicles. However, on the other hand, it is assumed that a successfully placed prosthesis could necrotize or change its placement in respect of the stapes long process in the long-term, which, consequently, bends the stapes prosthesis and changes the piston placement of the prosthesis. Many authors [8,17,18,19,20] have emphasized that incus necrosis is amongst the most common causes of stapedectomy failure. Theories for why this occurs vary but include overly zealous crimping, loss of blood supply, or a poorly fitted prosthesis, causing inflammation and bone erosion over time [8,17,18,19,20].

In this article, two issues were revealed concerning the stapes prosthesis placement and the necessity of a new, more adapted, stapes prosthesis:

- Over time, when the fixed prosthesis is in contact (naturally or compulsory, e.g., the moving bottom part slips out of the cochleostomy and is constrained elsewhere), the contact interaction changes, due to which the prosthesis loses its functions.
- When the ear is cut open during the stapes surgery, it can be seen that the incus long process is “anatomically imperfect” for the classic (standard) stapes prostheses.

A new modified stapes prosthesis (adjustable angled) was modelled to solve the previously presented problematics. The same statement was highlighted by Massimillia et al. [17] that during the revision, piston replacement with the same type or angled type prosthesis, in cases of erosion or minimal long process incus necrosis, provided excellent hearing results.

Consequently, in the next chapter, two sets of numerical modal analyses are presented.

The first set of numerical analyses represents the dynamical behavior of the standard stapes prosthesis, according to the stapes prosthesis fixation. The second set of numerical analyses represents the dynamical behavior of the modified stapes prosthesis (mainly adapted for the incus long process and prosthesis positioning), according to the stapes prosthesis fixation.

## 2. Methods: The Eigenfrequency Analysis of Standard and Modified Stapes Prosthesis

In the previous chapter, an issue was raised that there have been observed clinical situations where the stapes prosthesis loses its ability to transmit mechanical vibrations in the long-term.

**Figure 4:**
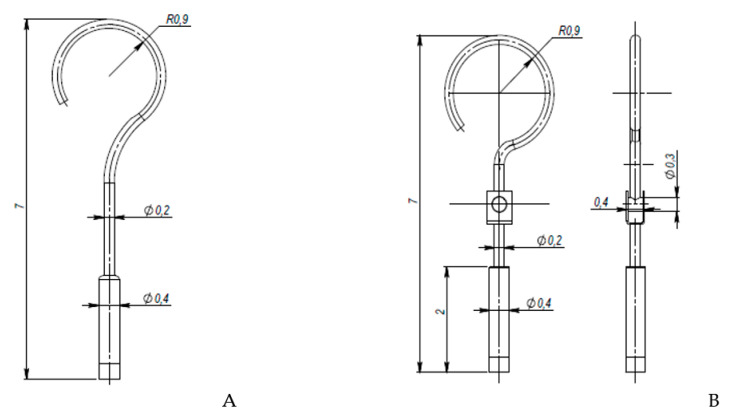
3D CAD designs of stapes prosthesis. (**A**) standard, (**B**) modified with cylindrical joint.

This research aims to evaluate the mechanical behavior for vibrations of the standard stapes prosthesis and a new modified stapes prosthesis. For this purpose, a standard stapes prosthesis with recommended measurements (diameter of 0.4 mm and 0.2 mm) has been modeled for the attachment to a footplate hole of 0.6 mm diameter cochleostomy. Accordingly, Figure 4 presents Computer Aided Design (CAD) 3D models of a stapes prosthesis (standard (**A**) and modified with joint (**B**)), modelled using SolidWorks^®^v Premium 2021 × 64 Edition software. CAD models were meshed with a high quality mesh. A standard stapes prosthesis (Figure 4A) contained 45,636 elements and 70,582 nodes. A modified stapes prosthesis with a cylindrical pin joint (Figure 4B) contained 48,402 elements and 76,172 nodes. In both cases, the bottom prosthesis cylinder of 0.4 mm diameter in the numerical analysis was made of Teflon and the remaining part (0.2 mm) of titanium alloy. Accordingly, the height of the prosthesis is 7 mm, and the radius of the fixing loop is 0.9 mm. Correspondingly, the mechanical properties of Teflon and the titanium alloy have been prescribed to the numerical model.

Finally, there were conducted two series of numerical analyses by changing the boundary conditions. Table 1 presents the calculation setup for eigenfrequency numerical analysis with boundary conditions, according to the standard stapes prosthesis placement and clinical situation. Correspondingly, 4 different numerical models were created, according to the boundary conditions:

1. Tight or target fixation of the fixing loop of the prosthesis and free movement of the piston part of the prosthesis. The boundary conditions of the free piston correspond to the case when in a cochleostomy, the thin scar tissues have not yet surrounded the prosthesis.
2. The over-constrained fixing loop of the prosthesis and free movement of the piston part of the prosthesis. The boundary conditions of the over-constrained fixing loop corresponded to the case when necrosis or hyperostosis progressed over the long process. The boundary conditions of the free piston correspond to the case when in a cochleostomy, the thin scar tissues have not yet surrounded the prosthesis.
3. Tight or target fixation of the fixing loop of the prosthesis. The boundary conditions represent the sliding of the piston along the cochleostomy.
4. The over-constrained fixing loop of the prosthesis and the over-constrained bottom of the prosthesis due to the malfunction of the prosthesis.

The previously detailed boundary conditions follow the hypothesis that postoperative tissues have outgrown the stapes prosthesis [13], and additionally, the fixation in the cochleostomy was taken into account.

As presented in Figure 3B, the prosthesis bends, and the piston placement of the prosthesis changes because of the change in the placement of the stapes long process and the prosthesis. Therefore, there was modelled a stapes-modified prosthesis with a joint to compensate for mechanical bending or adapt to the leaning angle of the incus long process. As presented in Table 1, correspondingly, the numerical analysis was performed according to the setup of boundary conditions, as previously detailed.

Table 2 presents the eigenfrequency analysis results and the 1st calculated mode of the standard prosthesis, according to the boundary conditions. The picture provided above of each presented mode shows the constraining type.

The results presented in Table 2 represent the dynamical behavior of standard stapes prostheses, according to the type of constraining. It can be deduced that the over-constrained standard prosthesis exceeds the speech frequency (the 1st mode was found at 7 kHz). Furthermore, it can be seen that in both cases (tight crimping—without and with bottom’s fixation) the 1st mode is found at 0.24 kHz and 0.51 kHz, respectively. Accordingly, this dynamical behavior will generate the movement of the loop along the long process. From a medical point of view, this situation with the standard prosthesis could lead to sulcus formation and necrosis of the long process of the incus.

Accordingly, Table 3 presents the results of the 1st calculated mode of the standard modified prosthesis with the cylindrical joint, according to the boundary conditions. The picture presented above of each calculated mode shows the constraining type. Here, it can also be seen that the over-constrained modified prosthesis exceeds the range of the speech frequencies (the 1st mode was found at 13 kHz).

Contrary to the standard prosthesis, the results of the dynamic behavior of the modified prosthesis show that the 1st mode exhibits vibrations of the thicker piston part and the junction with the piston. From a medical point of view, this kind of dynamic behavior of the angled prosthesis may allow avoidance of erosion of the long process of the incus.

## 3. Results and Discussion

The conducted numerical analysis provides new insights into the constraining of the prosthesis and its impact on improving the hearing after a surgical operation when the ossicular chain is recreated.

The success of middle ear reconstructive surgery depends on stable coupling between the prosthesis and residual ossicles [21]. Furthermore, it was mentioned by Eiber et al. [14] that a stable coupling between the prostheses and the remaining ossicular rudiments is a precondition for satisfactory hearing results. The fixation of the stapes prosthesis loop on the incus long process is influenced by the crimping of the loop [12,13]. Moreover, after surgery, connective tissue and mucosa may grow over the coupling area and thereby influence the sound transmission properties of the incus– prosthesis interface [13].

In the previous chapter, the results of the calculated first mode presented the differences in the dynamical behavior, according to the type of fixation of the prosthesis and its geometry. As it was proved by Sim et al. [13], the overgrowing tissue in the incus–prosthesis interface has only a small effect on the sound transmission properties. The numerical results of the impact of the loop constraining are presented in Table 2 and Table 3 (Case Study 1 and 2). Therefore, taking into account that the incus mobility is estimated in the range of frequencies up to 8 kHz, in the case study of the standard prosthesis, there has been observed a difference in Hz of the first exhibited mode by only 1% (Case Study 1 and 2, Table 2). Correspondingly, for the angled prosthesis, the difference of the first exhibited mode is 0.5%, according to the constraining of the loop (Case Study 1 and 2, Table 3).

**Figure 5:**
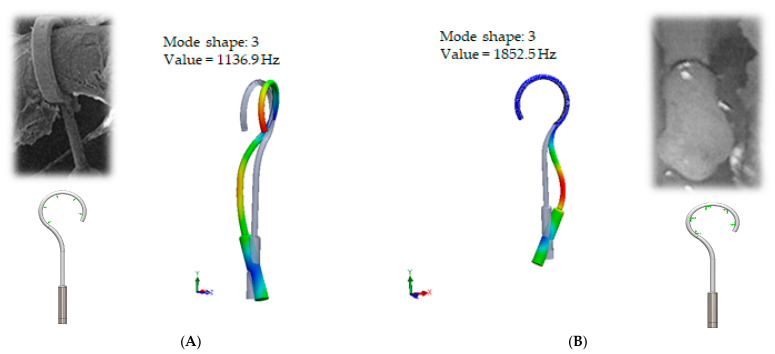
The results of modal analysis: the fixation of the loop of standard stapes prosthesis on the incus long process with free piston. (**A**) standard (target) fixation of the loop of prosthesis. (**B**) over constrained fixation of the loop of prosthesis (for example, in case of necrosis).

Consequently, other results of the numerical analysis, according to the fixation of the loop, are presented in Figure 5. As it can be seen, the third mode of the standard prosthesis constraining with target crimping is 1.14 kHz (Figure 5A), whereas the third mode of the over-constrained loop of the standard stapes prosthesis is 1.9 kHz (Figure 5B). Moreover, it has been found that this third mode exhibits the bending of the thick part of the piston, due to which the prosthesis tends to bend and contact boundary surfaces in the cochleostomy.

Finally, this analysis reveals that the over-constrained attachment of the loop of the prosthesis (free piston in the cochleostomy) impacts the bending of the prosthesis and causes erroneous positioning of the piston in the cochleostomy (see Figure 5B).

However, a completely loose piston in the cochleostomy is not possible in respect of physics. The real fixation of the prosthesis can/will change because in the auditory system, the biological material is interacting with the plastic/metal over time. The loop of the prosthesis can necrotize, and the scar tissue can surround it more or less in the cochleostomy.

**Figure 6:**
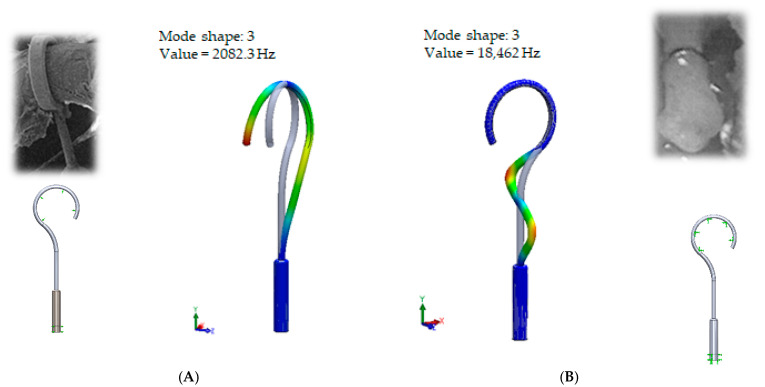
The results of modal analysis of fixing the standard stapes prosthesis on the incus long process and in cochleostomy. (**A**) target constraining of prosthesis, (**B**) over-constraining of the prosthesis.

Thus, the results when the prosthesis hugs the incus long process and at the same time the bottom part of the prosthesis is constrained in the cochleostomy are provided in Figure 6.

In Figure 6A presents standard/target constraining of the standard prosthesis: tight crimping of the long process and sliding of the bottom part of the prosthesis. Here, it can be observed that the third mode exhibits twisting mainly around the axis of the long process.

In Figure 6B presents the over-stretched prosthesis. In this case, the standard prosthesis is constrained as a cord due to the over-constrained crimping of the stapes long process and tight attachment to the footplate (when the prosthesis bends, it loses mobility, as the side surface is constrained in the cochleostomy). It can be observed that this third mode exhibits the twisting in the middle part of the prosthesis.

From the numerical results provided in Figure 6, it can be seen that the over-constrained bottom of the stapes prosthesis and over-stretching of the fixing loop of the prosthesis actually corresponds to a complicated clinical case. In this case, the prosthesis is stimulated as a cord, and it can be seen that the first stimulating mode exceeds the speech frequencies (2 kHz). This would explain why after some time, the prosthesis “stops working”. The first mode, as well as the third mode, of the standard stapes prosthesis exhibits the bending and twisting of the middle part of the standard stapes prosthesis (Table 2 and Figure 6).

**Figure 7:**
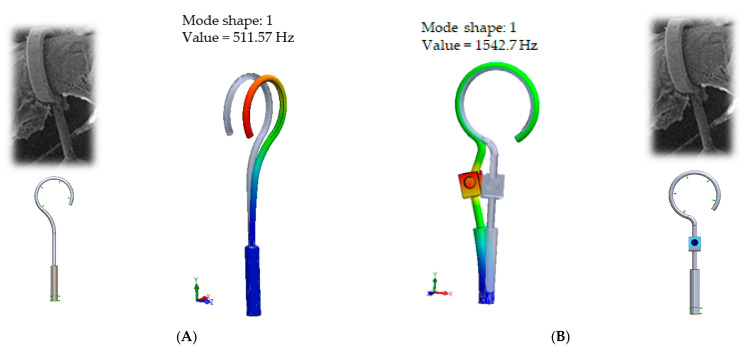
The results of modal analysis of target fixing of prosthesis. (**A**) standard stapes prosthesis, (**B**) modified stapes prosthesis.

When comparing the first mode of the standard stapes prosthesis and the modified stapes prosthesis according to ideal/target constraining, the first mode was found at 0.51 kHz frequency for the standard prosthesis and accordingly at 1.54 kHz frequency for the modified prosthesis (Figure 7). Here, in the standard prosthesis, the first mode exhibits the movement around the long process, and in the adjustable angled prosthesis, the first mode exhibits the bending in the part with the joint. It could be stated that it is an advantage of the modified stapes prosthesis because if the loop or thick part of the prosthesis were not stimulated, a sulcus in the incus long process would not be formed due to the continuous stimulation, as well as in a cochleostomy, where the movement of the prosthesis should remain even. However, the joint of the modified prosthesis would allow for the prosthesis to adapt to various placements in respect of the stapes long process and the prosthesis.

## 4. Conclusions

The presented research work revealed a study of the dynamic behavior of a modified adjustable stapes prosthesis, which may allow avoidance of anatomical disturbances and instability of the prosthesis in the long-term due to the necrosis/hyperostosis. The dynamic behavior of the proposed modified prosthesis has been obtained in comparison to the standard stapes prosthesis that is used in the case of otosclerosis.

The conducted modal analysis of the standard and modified stapes prosthesis in respect of its constraining has shown that the over-constrained standard prosthesis exceeds the speech frequency (the first mode was found at 7 kHz). Moreover, it has been found that the dynamic behavior of the standard stapes prosthesis will generate movement of the loop along the long process. From a medical point of view, this situation with the standard prosthesis could lead to sulcus formation and necrosis of the long process of the incus. From a scientific point of view, this finding could explain why after some time, the prosthesis “stops working”, and the patient needs revision surgery.

Summing up, even though there is a variety of different prostheses on the market, this research reveals that existing prostheses do not correspond to all clinical situations when aiming to regain hearing by reconstructing the ossicular chain.

The presented findings of the case study of the stapes prosthesis constraining in the case of otosclerosis reveal the idea that some progress of the interaction of biological material and plastic/metal could be avoided by adopting an appropriate stapes prosthesis.

## Figures and Tables

**Table 1:** The setup of stapes prosthesis constraining and boundary conditions for eigenfrequency analysis.

Numerical Description of the Study	Clinical Description of the Case Study			
	**(1) Loop: Tight or Target Crimping** Piston: Free in Cochleostomy *	**(2) Loop: Over Constrained** **Piston: Free in** Cochleostomy *	**(3) Loop: Tight or Target Crimping** **Piston: Sliding in Cochleostomy**	**(4) Loop: Over Constrained** **Piston: Over Constrained** **due to Malfunction**
Clinical visualization of crimping of the loop of prosthesis Source: adapted from Huber et al. [12]	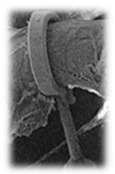	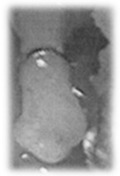	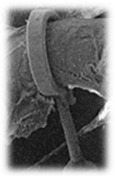	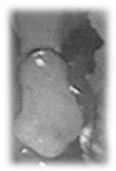
Boundary conditions **	Loop: 1T along axis of long process Piston: no constrains	Loop: 6 DOF eliminated Piston: no constrains	Loop: 1T along axis of long process Piston: 1 T along the axis of piston	Loop: 6 DOF eliminated Piston: 6 DOF eliminated
3D numerical model of standard stapes prosthesis	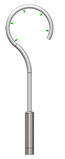	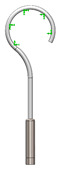	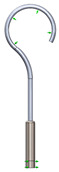	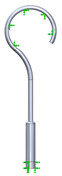
3D numerical model of modified standard stapes prosthesis with cylindrical pin connector	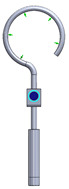	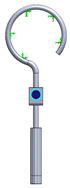	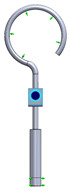	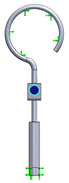

* In this case, the boundary conditions correspond to the case when in cochleostomy, the thin scar tissue have not yet surrounded the prosthesis. ** 6 DOF—6 Degrees of Freedom.

**Table 2:** The results of eigenfrequency analysis of standard prosthesis: the 1st mode and the eigenfrequencies (Hz) of first 4 modes.

The Type of Constraining of Standard Prosthesis			
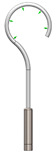	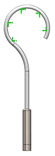	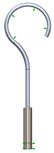	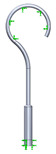
1	2	3	4
The 1st mode of standard prosthesis, according to the boundary conditions			
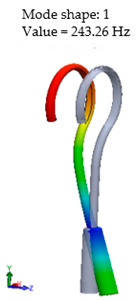	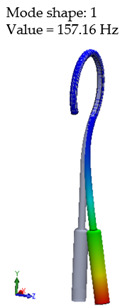	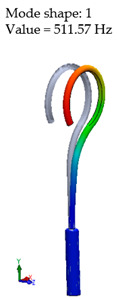	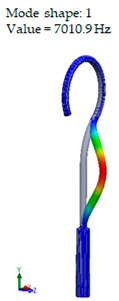
The first 4 eigenfrequencies (Hz), according to the modes			
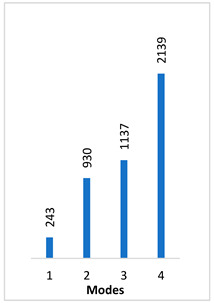	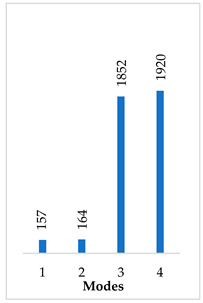	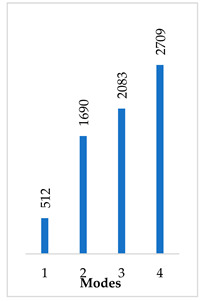	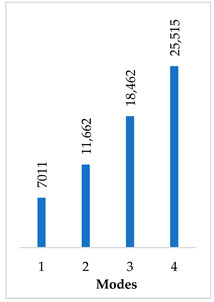

**Table 3:** The results of eigenfrequency analysis of modified standard prosthesis: the 1st mode and the eigenfrequencies (Hz) of first 4 modes.

The Type of Constraining of Modified Standard Prosthesis			
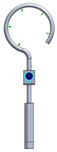	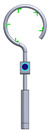	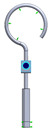	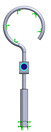
1	2	3	4
The 1st mode of standard prosthesis, according to the boundary conditions			
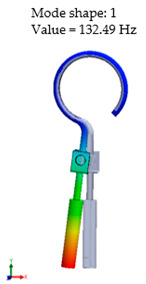	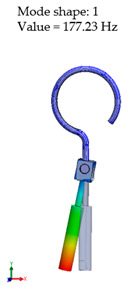	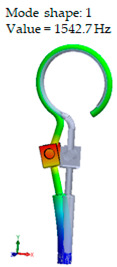	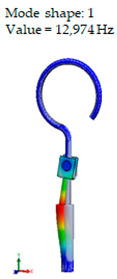
The first 4 eigenfrequencies (Hz), according to the modes			
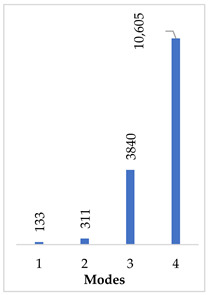	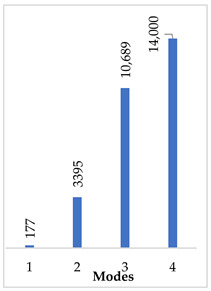	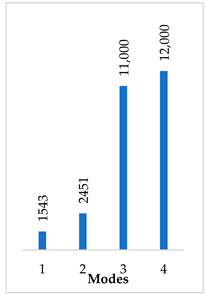	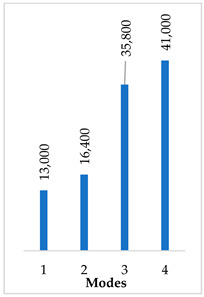

## Data Availability

The data presented in this study are available on request from the corresponding author.
